# A pilot study exploring the association of bronchial bacterial microbiota and recurrent wheezing in infants with atopy

**DOI:** 10.3389/fcimb.2023.1013809

**Published:** 2023-02-15

**Authors:** Wei Tang, Lei Zhang, Tao Ai, Wanmin Xia, Cheng Xie, Yinghong Fan, Sisi Chen, Zijin Chen, Jiawei Yao, Yi Peng

**Affiliations:** ^1^ Respiratory Department, Chengdu Women’s and Children’s Central Hospital, School of Medicine, University of Electronic Science and Technology of China, Chengdu, China; ^2^ School of Clinical Medicine, Chongqing Medical and Pharmaceutical College, Chongqing, China

**Keywords:** 16S rRNA, wheezing, infants, atopy, microbiome

## Abstract

**Background:**

Differences in bronchial microbiota composition have been found to be associated with asthma; however, it is still unclear whether these findings can be applied to recurrent wheezing in infants especially with aeroallergen sensitization.

**Objectives:**

To determine the pathogenesis of atopic wheezing in infants and to identify diagnostic biomarkers, we analyzed the bronchial bacterial microbiota of infants with recurrent wheezing and with or without atopic diseases using a systems biology approach.

**Methods:**

Bacterial communities in bronchoalveolar lavage samples from 15 atopic wheezing infants, 15 non-atopic wheezing infants, and 18 foreign body aspiration control infants were characterized using 16S rRNA gene sequencing. The bacterial composition and community-level functions inferred from between-group differences from sequence profiles were analyzed.

**Results:**

Both α- and β-diversity differed significantly between the groups. Compared to non-atopic wheezing infants, atopic wheezing infants showed a significantly higher abundance in two phyla (*Deinococcota* and unidentified bacteria) and one genus (*Haemophilus*) and a significantly lower abundance in one phylum (*Actinobacteria*). The random forest predictive model of 10 genera based on OTU-based features suggested that airway microbiota has diagnostic value for distinguishing atopic wheezing infants from non-atopic wheezing infants. PICRUSt2 based on KEGG hierarchy (level 3) revealed that atopic wheezing-associated differences in predicted bacterial functions included cytoskeleton proteins, glutamatergic synapses, and porphyrin and chlorophyll metabolism pathways.

**Conclusion:**

The differential candidate biomarkers identified by microbiome analysis in our work may have reference value for the diagnosis of wheezing in infants with atopy. To confirm that, airway microbiome combined with metabolomics analysis should be further investigated in the future.

## 1 Introduction

Wheezing is a common condition observed in early childhood. It has been reported that approximately 50% of infants experience wheezing before the age of 2 years (Zanobetti et al., 2022), and approximately 1/3 of preschool-age children experience recurrent wheezing (Ünal et al., 2017). Many infants who experience wheezing show allergic sensitization. Based on the asthma prediction index, atopic infants with recurrent wheezing are at high risk for developing asthma (Rodríguez-Martínez et al., 2017; Schmidt et al., 2019). Pediatric asthma is associated with a high incidence of chronic diseases in children. However, the underlying mechanism remains poorly understood.

Pathogenic respiratory infections and allergen sensitization are considered the most common causes of wheezing in infants (Weiss, 2008). Large amounts of microbiota are present throughout the respiratory tract, and the composition of microbiota differs in richness and diversity between individuals (Man et al., 2017), which is the cause of occurrence and development of diseases (Budden et al., 2019). The phenotypic features of asthma, such as airway hyper-responsiveness, asthma control, and transcriptional response to steroids, correlate with bronchial microbial composition (Huang et al., 2015). Airway microbiota may provide predictive value for asthma development in infants (Shah & Bunyavanich, 2021). Most studies of the role of airway microbiota in childhood asthma and wheezing have focused on the upper respiratory tract (Pérez-Losada et al., 2018; Toivonen et al., 2019; McCauley et al., 2019). The influences of the lower respiratory tract bacterial community in wheezing infants, especially in those with atopy, are still being established.

In this study, we aimed to evaluate the differences in microbiota in bronchoalveolar lavage fluid (BAL) between recurrent wheezing and non-wheezing infants using 16S rRNA sequencing technology. Subgroup analyses were used to further confirm the relationships between lower respiratory microbiota and atopy. The study groups did not differ in other confounding factors. Tools for bacterial identification, microbial community biodiversity assessment, and functional predictions were used.

## 2 Materials and methods

### 2.1 Subjects and BAL collection

This study was conducted observationally. Infants were recruited from the Chengdu Women’s and Children’s Central Hospital between January 2019 and December 2020. This study was approved by the Ethics Review Board of Chengdu Women’s and Children’s Central Hospital 2016 (22). Written informed consent was obtained from the guardians of all participants. Included in the study were 30 infants who met the following criteria: 1) aged 1–3 years, 2) wheezing occurrence ≥2 times, 3) inhaled corticosteroids and antibiotics not administrated within one week, and 4) diagnosed with wheezing by a physician specializing in pediatric pulmonology. Of the 30 infants, 15 were found to be atopic. Atopy was defined based on skin prick test evidence (wheal diameter >25% induced by histamine) of sensitivity to ≥1 of 12 aeroallergens (allergen skin prick liquids; Aroger Company, Germany). Additionally, 18 children aged 1–3 years who underwent bronchoscopy due to foreign body aspiration within 24 h of sample collection were used as control subjects. Infants > 36 months of age with congenital heart diseases, immunodeficiency, bronchopulmonary dysplasia, cystic fibrosis, or neuromuscular disorders were excluded. Bronchoalveolar lavage (BAL) was performed using a fiberoptic bronchoscope through the airway *via* a laryngeal mask. BAL samples were collected from wheezing infants and controls, and centrifuged at 14000 × g for 10 min. The pellets were resuspended in 500 μL of phosphate buffered saline (PBS) and stored at -80°C until DNA extraction.

### 2.2 DNA extraction and 16S rRNA gene sequencing

Total genomic DNA was extracted from the samples using the CTAB/SDS method, and 2% agarose gel electrophoresis was used to detect the purity and concentration of DNA. 16S rRNA gene amplification were performed targeting the V3-V4 hypervariable region (Forward primer 5′- CCTAYGGGRBGCASCAG -3′and Reverse primer 5′- GGACTACNNGGGTATCTAAT -3′). Then, 2% agarose gel electrophoresis was used to detect PCR products. Qualified barcoded DNA amplicons were purified, quantified, and pooled to construct the sequencing library using TruSeq^®^ DNA PCR-Free Sample Preparation Kit (Illumina, California, USA). The constructed library was quantified using Qubit and Q-PCR, and then sequenced on an Illumina NovaSeq 6000 (Illumina, California, USA) platform to generate pair-ended 250 bp reads.

### 2.3 Data processing

Quality filtering, denoising, and chimera removal of all raw data from sequencing were performed using the Quantitative Insights Into Microbial Ecology (QIIME) platform (Caporaso et al., 2010). In brief, the barcode sequence and PCR amplification primer sequence of each sample’s raw data was split. FLASH (V1.2.7) was used to splice the reads of each sample to obtain the splicing sequence (Raw Tags). And then referring to QIIME’s (V1.9.1) Tag quality control process, the following operations were performed: a) Tags truncated: Raw Tags were truncated from the first low-quality base site where the number of consecutive low-quality bases (the default quality threshold is ≤19) reached the set length (default length value); b) Tags filtered: Tags whose consecutive high-quality base lengths were less than 75% of the Tag length were filtered out; and c) Chimera sequence removed: The tag sequence was compared with the species annotation database to detect the chimera sequence, and the chimera sequence was removed to obtain the final valid data (Effective Tags) for the next step of analysis. Uparse algorithm was performed to cluster the Effective Tags. A 97% sequence homology cut-off was used to define operational taxonomic units (OTUs), which generally approximate the differences in 16S sequences between bacterial species. For clustered OTUs, taxonomic information was annotated using the Silva database (set the threshold to 0.8~1). The main microbiota was identified at the phylum and genus levels. QIIME software (version 1.9.1) was used to calculate alpha and beta diversity, and alpha and beta diversity index between groups was analyzed using R software (wilcox test). FastTree (Price et al., 2009) was used to build the phylogenetic tree. R software (version2.15.3) was employed to perform species abundance cluster heatmap and Metastats analyses of phyla and genera. Linear discriminant (LDA) effect size (LEfSe) analysis was used to determine microbial differences between groups. Random forest modelling was performed using R software (version 3.2.1). Phylogenetic Investigation of Communities by Reconstruction of Unobserved States (PICRUSt2) was adopted to predict microbial functions (Langille et al., 2013; Douglas et al., 2020), and grouping of predicted pathways was performed using the Kyoto Encyclopedia of Genes and Genomes (KEGG) (Kanehisa & Goto, 2000), at hierarchy level 3.

### 2.4 Statistical analysis

Quantitative data are expressed as mean values with standard deviations (SD), and enumerated data are presented as frequencies. The enumeration data were analyzed by Pearson’s Chi-square test. Student’s t-test or Wilcoxon signed-rank test (agricolae package) was used to compare continuous parametric or non-parametric variables, respectively. The Benjamini-Hochberg method that controls false discovery rate for multiple testing was used. The R statistical software package (R version 2.15.3) was used to perform all statistical analyses and graphics. Statistical significance was set at p<0.05.

## 3 Results

### 3.1 Participant characteristics

In total, 30 recurrently wheezing infants (wheezing group, Whz, n=30) were enrolled. Wheezing infants were separated into atopic wheezing (Aw, n=15) and non-atopic wheezing (nAw, n=15) groups. Also enrolled were 18 infants with foreign body aspiration (control group, Con, n=18). Clinical demographics of the participants are presented in **Table 1**. There were no significant differences in age, sex, Cesarean section status, birth weight, premature birth status, blood WBC count, neutrophil count, or eosinophil proportion between the wheezing and control groups. Atopy differed significantly between the two groups. Considering the effects of atopy on airway microbiota, we conducted a subgroup analysis based on whether wheezing infants were atopic or not to further confirm the relationship between respiratory microbiota and atopy. The detailed characteristics of the Aw and nAw groups are shown in **Table 2**.

**Table 1:** Clinical characteristics of all the enrolled participants.

	Wheezing group (n=30)	Control group (n=18)	P value
Age (month)	14.80±2.92	18.61±1.43	0.34
Gender (M/F)	21/9	11/7	0.53
Atopy (Y/N)	15/15	3/15	0.02
Cesarean (Y/N)	17/13	10/8	0.94
Birth weight (kg)	2.98±0.88	3.11±0.81	0.35
Premature (Y/N)	5/25	1/17	0.39
WBC counts (×10^9^/L)	10.22±0.69	10.69±0.75	0.69
Netrophils (%)	42.28±3.01	32.13±5.49	0.85
Eosinophils (%)	2.06±0.33	2.06±0.34	0.99

**Table 2:** Clinical characteristics of recurrent wheezing infants with and without atopy.

	Atopic wheezing (n=15)	non-Atopic wheezing (n=15)	P value
Age (month)	13.67±1.22	15.93±5.80	0.71
Gender (M/F)	11/4	10/5	0.69
Cesarean (Y/N)	7/8	10/5	0.27
Birth weight (kg)	2.92±0.11	3.04±0.14	0.51
Premature (Y/N)	3/12	2/13	0.62
WBC counts (x10^9^/L)	10.55±0.89	9.89±1.08	0.64
Netrophils (%)	48.03±4.19	36.54±3.91	0.06
Eosinophils (%)	2.21±0.50	1.91±0.44	0.65
Ti/Te	0.56±0.02	0.57±0.03	0.65
TPTEF/TE (%)	18.35±1.14	19.47±2.23	0.66
VPEF/VE (%)	20.75±0.77	22.53±1.71	0.35

Ti/Te, the ratio of inspiratory time and expiratory time.

TPTEF/TE, time to peak tidal expiratory flow as a proportion of expiratory time.

VPEF/VE, volume to peak tidal expiratory flow as a proportion of exhaled volume.

### 3.2 Microbiota in BAL

#### 3.2.1 Estimation of sequencing depth

After quality control filtering, a total of 2,956,642 high-quality tags were obtained, and the average tags of wheezing and control groups were 60,590 and 63,274, respectively. A total of 4,873 OTUs with at least 97% sequence homology were identified by clustering. Clustered OTUs were annotated with Silva138, and 1,843 (37.82%) OTUs were annotated at the genus level.

**Figure 1:**
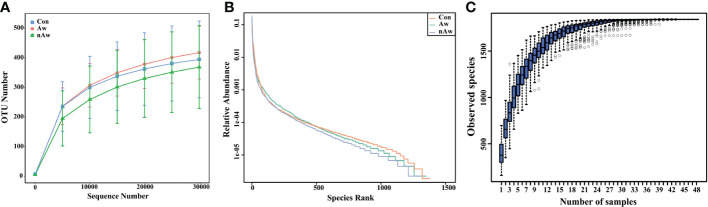
Estimation of sample depth in the Aw, nAw, and Con groups. **(A)** Rarefaction curves. **(B)** Rank Abundance curves. **(C)** Species Accumulation Boxplots. Aw, atopic wheezing group; nAw, non-atopic wheezing group; Con, foreign body aspiration infants as controls.

The rarefaction curve reflects the rationality of the sequencing data. As shown in **Figure1A**, the rarefaction curves gradually tended to be flat, indicating a reasonable amount of sequencing data in this study. The rank abundance curve indicates the richness and evenness of the species in the sample. The horizontal width of the curve is related to species richness, whereas the vertical smoothness is related to evenness. As shown in **Figure1B**, the rank abundance curves indicated that the richness and evenness of each sample group were high. A species accumulation boxplot was used to determine whether the sample size was sufficient. As shown in **Figure 1C**, the species accumulation boxplot gradually reached the platform stage with increasing sample size, indicating that the sample size was sufficient.

#### 3.2.2 Alpha-diversity and beta-diversity

**Figure 2:**
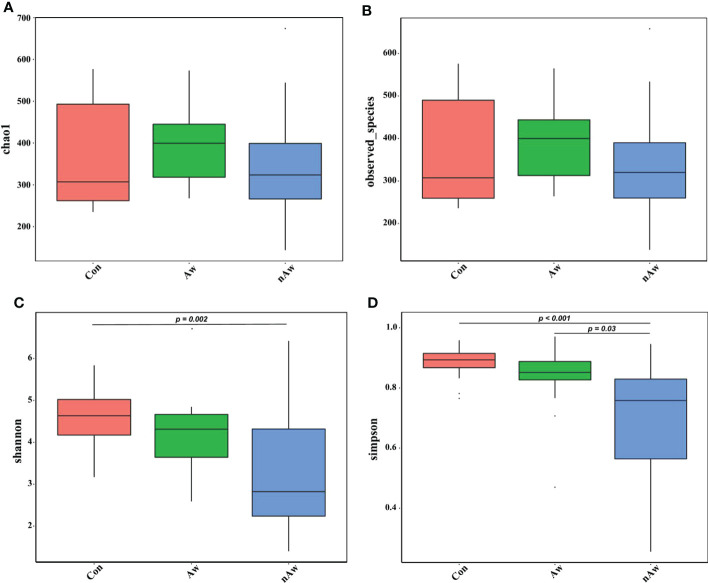
Alpha-diversity analysis of microbiome in BAL. **(A)** Chao1 index statistics. **(B)** Observed_species index statistics. **(C)** Shannon index statistics. **(D)** Simpson index statistics. Wilcox test was used. Aw, atopic wheezing group; nAw, non-atopic wheezing group; Con, foreign body aspiration infants as controls.

Alpha diversity was adopted to analyze the richness (number of taxonomic groups) and evenness (distribution of abundances of the groups) within the microbial communities of the same group. Chao1, observed species, Shannon, and Simpson indexes were evaluated for each sample. The Shannon indices analyzed *via* Wilcoxon test showed significant differences between the nAw and Con groups. Simpson indices showed significant differences between nAw and Con and between Aw and nAw (**Figure 2**).

**Figure 3:**
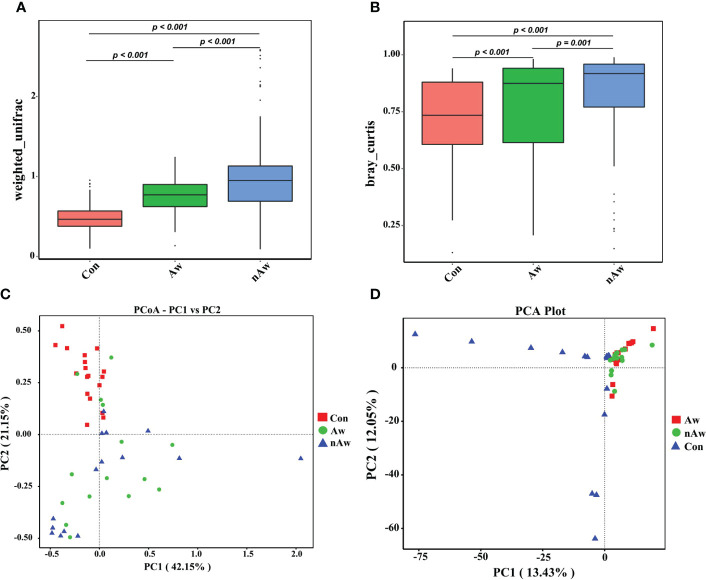
Beta-diversity analysis of microbiome in BAL. **(A)** Beta diversity index based on Weighted Unifrac, **(B)** Beta diversity index based on Bray_Curtis, **(C)** Display of Principal Co-ordinates Analysis (PCoA) plots of the samples in two-dimension based on weighted unifrac (PC1 = 42.15%, PC2 = 21.15%), **(D)** Display of Principal Component Analysis (PCA) of the samples in two-dimension (PC1 = 13.43%, PC2 = 12.05%). Wilcox test was used. Aw, atopic wheezing group; nAw, non-atopic wheezing group; Con, foreign body aspiration infants as controls.

The beta diversity index based on the weighted Unifrac and Bray_Curtis metrics showed significant differences among the three groups (**Figures 3A, B**). The Wilcoxon test was used to calculate differences. Principal coordinate analysis (PCoA) plots based on weighted analysis and principal component analysis (PCA) were used for data visualization (**Figures 3C, D**).

### 3.3 Distribution of microbial taxonomic composition in wheezing infants

**Figure 4:**
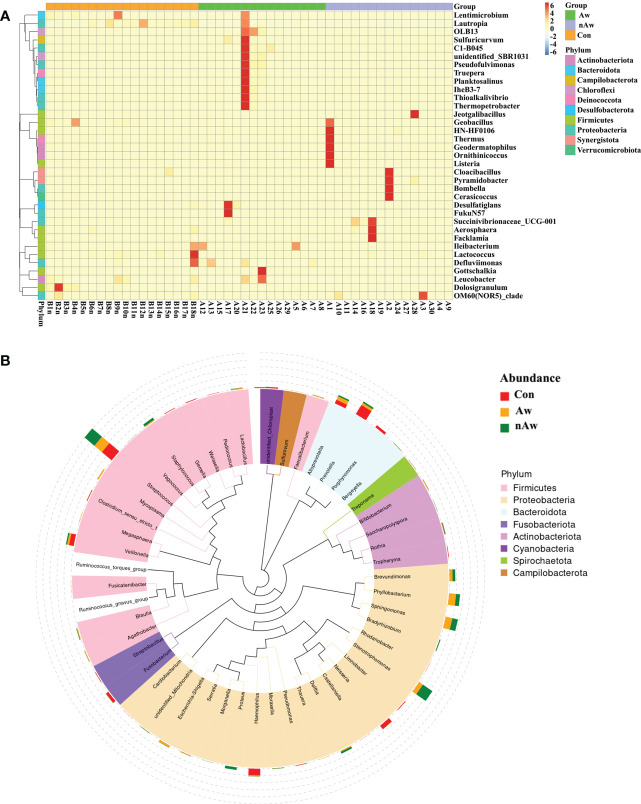
Heatmap and Phylogenetic tree at genus level. **(A)** Cluster heatmap. The plot depicts the relative abundance of each genus (x-axis) within each sample (y-axis). **(B)** Phylogenetic tree. The branch and fan colors indicate their corresponding gates, and stacked histograms outside the fan rings indicate the abundance distribution. Aw, atopic wheezing group; nAw, non-atopic wheezing group; Con, foreign body aspiration infants as controls.

To visually examine the similarities and differences in community composition of each group at the genus level, abundance information of the top 35 genera was obtained for clustering *via* the maximal ranking method, and a heat map was generated from the species annotation and abundance information. Cluster analysis *via* heatmap showed that the bacterial composition at the genus level differed among the three groups (**Figure 4A**). In order to study the phylogenetic relationships of species at the genus level, representative sequences of the top 50 genera were obtained by multiple sequence alignment, and phylogenetic trees were constructed based on the representative sequences. (**Figure 4B**).

### 3.4 The distribution of taxa at phylum and genus levels in wheezing infants

**Figure 5:**
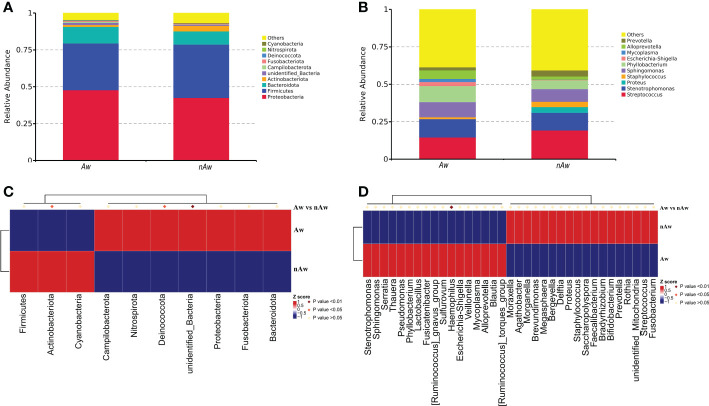
The distribution of taxa in phylum and genus levels of Aw and nAw. **(A)** composition of microbiome at the phylum level. **(B)** composition of microbiome at the genus level. **(C)** the statistical results of top 10 phylum. **(D)** the statistical results of top 35 genus. Aw, atopic wheezing group; nAw, non-atopic wheezing group.

Based on the species annotation results, the top 10 species with the highest abundance in the phylum and genus levels were selected from the atopic and non-atopic wheezing groups, and a relative abundance histogram was generated. Proteobacteria, Firmicutes, and Bacteroidetes were the most abundant phyla in the airway microbiota (**Figure 5A**), whereas *Streptococcus, Stenotrophomonas, Sphingomonas*, and *Phyllobacterium* were the dominant genera in both groups (**Figure 5B**). Deinococcota and unidentified bacteria phyla were more abundant in Aw (p<0.05 and p<0.01, respectively). **Figure 5C**), while Actinobacteria was more abundant in nAw (p<0.05, **Figure 5C**). At the genus level, *Haemophilus* was significantly enriched in Aw compared with nAw (p<0.05, **Figure 5D**).

### 3.5 Linear discriminant analysis in wheezing infants

**Figure 6:**
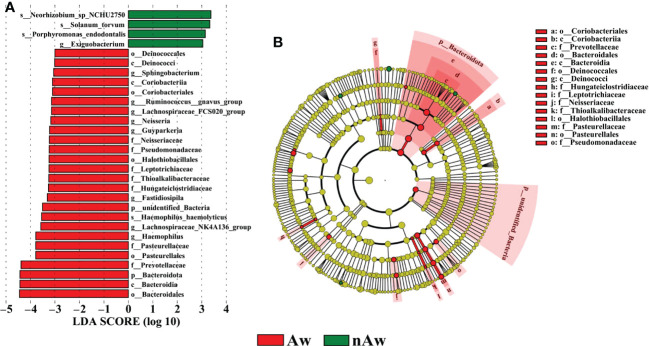
Linear discriminant analysis (LDA). **(A)** Histogram of the LDA scores (>3) computed for bacterial taxa differentially abundant between Aw and nAw. The size of each node represents the LDA score. **(B)** Bacterial taxa that were differentially abundant in the group visualized using a cladogram. The size of each node represents their relative abundance. Significantly discriminant taxon nodes are colored and branch areas are shaded according to the highest-ranked variety for that taxon. Aw, atopic wheezing group; nAw, non-atopic wheezing group.

To identify differences in bacterial taxa composition between Aw and nAw, LEfSe analysis was performed. Based on LDA scores of > 3.0, two phyla, three classes, five orders, seven families, eight genera, and one species were highly abundant in Aw, whereas one genus and three species were significantly more abundant in nAw. Bacteroidales, Bacteroidia, and Bacteroidota were significantly abundant in Aw, whereas Neorhizobium was more abundant in nAw (p<0.05, **Figure 6**).

### 3.6 Predictive modeling of the airway microbial profile in wheezing infants

#### 3.6.1 Random forest

**Figure 7:**
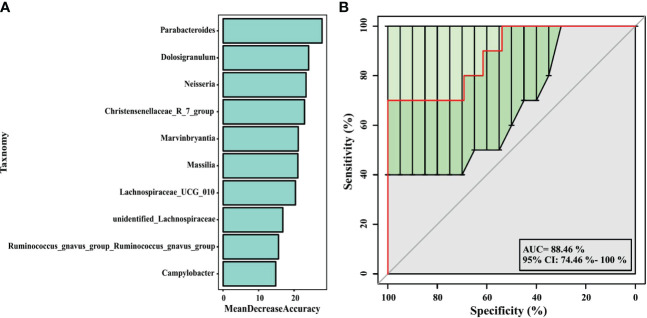
Prediction model of the airway microbiota for atopic wheezing status based on the genus-level relative abundances using random forests. **(A)** variable importance ranking chart, Mean Decrease Accuracy measures the degree to which the prediction accuracy of a random forest decreases by changing the value of a variable to a random number. A higher value indicates that the variable is more important. **(B)** ROC curve of the atopic wheezing model using 10 discriminatory genus. AUC, the area under the curve; ROC, receiver operating characteristic.

In our analysis, a random forest prediction model of ten genera was constructed using OTU-based features. In random forest, the sample dataset is divided into two parts at a ratio of 75:25, using 75% of the data to train the machine learning algorithm, and the remaining 25% for independent testing. Then, significant genera were selected using mean decrease in accuracy (**Figure 7A**). 10-fold cross-validation was conducted for the model, and receiver operating characteristic (ROC) curves were used to score the predictive power. The area under the curve (AUC) was 0.8846 (95% CI: 0.745−1.000) (**Figure 7B**), which indicated the airway microbiota diagnostic potential in wheezing infants. We observed that in the model, the 10 significant genera were *Parabacteroides, Dolosigranulum, Neisseria, Christensenellaceae_R_7_group, Marvinbryantia, Massilia, Lachnospiraceae_UCG_010, unidentified_ Lachnospiraceae, Ruminococcusgnavus_group_Ruminococcusgnavus_group*, and *Campylobacter*. These may serve as biomarkers to identify infants most likely to have atopic wheezing.

#### 3.6.2 Functional prediction

**Figure 8:**
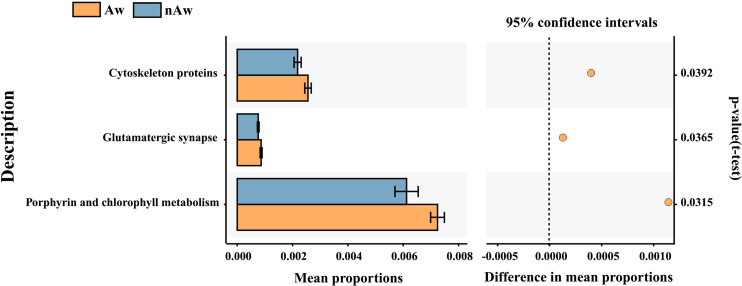
Predicted functional. Predicted functional differences between Aw and nAw microbiomes. A total of three metabolic pathways differed significantly between Aw and nAw infants. Pathways that were more abundant in Aw are on the positive side (orange circle with 95% confidence interval). Mean proportions are shown in stacks for Aw (orange) and nAw (blue). Differences in mean proportions: mean proportion in Aw minus mean proportions in nAw. Tests were conducted at KEGG hierarchical level 3, which included 294 pathways present in all samples. Aw, atopic wheezing group; nAw, non-atopic wheezing group.

PICRUSt2, a method to predict the functional potential of bacterial community using 16S rRNA amplicon data based on KEGG database, was used to evaluate the functional differences of microbiomes between Aw and nAw. 294 metabolic pathways were discovered in all samples (N = 294 pathways), and three metabolic pathways, namely glutamatergic synapse, cytoskeleton proteins, and porphyrin and chlorophyll metabolism, were significantly different between the two groups by statistical analysis metagenomic profiles (STAMP) test (**Figure 8**).

## 4 Discussion

In recent years, human lifestyles have changed to be largely indoors, and the number of infants exposed to environmental microbes has decreased. However, the annual incidence of allergic and wheezing diseases has increased. Atopic infants with recurrent wheezing are more likely to develop asthma, but the reason for this is unclear. The airway microbiota has been proposed to be linked to various pediatric respiratory diseases. Studies have reported that specific patterns of respiratory microbial colonization are associated with the severity of viral respiratory infections (Alba et al., 2021) and the phenotypic features of asthma (Durack et al., 2017a). Therefore, in-depth studies of the lower airway microbiota and its functional characteristics in infants with recurrent wheezing, especially atopic wheezing, are necessary. In this study, we collected BAL specimens from infants with recurrent wheezing and foreign body controls. Through high-throughput 16S rRNA sequencing, differences in the diversity, richness, composition, and function of the lower airway microbiome were compared. Our findings show diversity, compositional, and predicted functional differences in the bronchial bacterial microbiota between Aw infants and nAw infants.

The functional abundance of the microbiome depends on the individual and interactions between the environment and microbial community (Orland et al., 2019). Airway microbiota may be involved in the maturation and maintenance of respiratory physiology and immune homeostasis (Man et al., 2017). We found that the microbiota in BAL from both atopic and non-atopic wheezing infants was mainly composed of Proteobacteria, Firmicutes, and Bacteroidetes at the phylum level, and *Streptococcus*, *Stenotrophomonas*, *Sphingomonas*, and *Phyllobacterium* at the genus level. It is reported that, compared to healthy controls, asthmatic patients have higher microbiome diversity and altered composition, more Proteobacteria, and fewer Bacteroidetes (Sverrild et al., 2017). Another study (Hilty et al., 2010) found that the composition of the Proteobacteria phylum was associated with airway disease in both asthmatic and chronic obstructive pulmonary disease (COPD) patients. Currently, the genus *Stenotrophomonas* consists of four species, of which only one (*S. maltophilia*) is known to cause human infection (Coenye et al., 2004). In recent years, the human pathogen *S. maltophilia* has spread globally, and does not usually infect healthy hosts, but it has been found to be associated with high morbidity and mortality in severely immunocompromised individuals (An & Berg, 2018). Berdah et al. reported that *S. maltophilia* appeared to be a marker for cystic fibrosis lung disease severity (Berdah et al., 2018). Patients with asthma treated with inhaled corticosteroids had a higher abundance of Sphingomonas than patients without (Huang et al., 2021). A study of swine lung microbiota and their potential relationship with lung lesions found that *Sphingobium* and *Phyllobacterium* were the most abundant microbes in severely diseased lungs (Huang et al., 2019).

When bacterial taxa composition differences were compared, the results showed that the number of *Haemophilus* was significantly increased in atopic wheezing infants. This result is consistent with the findings of other authors. Durack et al. reported that haemophilus were uniquely enriched in adult asthmatic subjects, and the baseline compositional of the bacterial microbiota were linked to steroid-responsiveness (Durack et al., 2017a). The higher abundance of *Haemophilus* in the respiratory microbiota may regulate airway inflammation during severe RSV bronchiolitis in infancy, potentially contributing to the subsequent development of recurrent wheezing in childhood (Zhang et al., 2020).Current evidence suggests that the presence of specific microbes in the airways, particularly *Streptococcus*, *Haemophilus*, and *Moresila*, may shape local immune responses and alter the severity and presentation of airway inflammation (Ver Heul et al., 2019). Microbiomes dominated by *Haemophilus* have been shown to be associated with severe diseases and frequent recurrences (Richardson et al., 2019). Microbiomes in sputum are associated with the clinical and inflammatory phenotypes of COPD. Decreased microbiome diversity due to *Haemophilus* dominance is associated with increased neutrophil-associated protein profiles and an increased risk of mortality (Dicker et al., 2021). On the contrary, Wu et al. reported different results. They found that persistent wheezing infants with wheezing history had a lower abundance of Haemophilus compared with those without wheezing history (Wu et al., 2021). Differential results varied between existing studies, which may be due to heterogeneity between studies, such as sample size, study area, patient age, and clinical features.

Random forest models can be used to screen potential novel biomarkers. In this study, we constructed a random forest predictive model for the top 10 genera based on OTU-based features. The AUC was > 0.6, indicating that airway microbiomes have diagnostic value for distinguishing atopic wheezing infants from non-atopic wheezing infants. This indicates that the distinguishing bacteria can be used as candidate biomarkers. Among the top ten genera, *Parabacteroides* was the most significant marker for predicting atopic wheezing. Parabacteroides is a gram-negative anaerobic bacterium. At present, there are 15 species in the genus, of which 10 are “validly named” and 5 are “not validly named” according to the list of prokaryotic names with Standing in Nomenclature. The genus *Parabacteroides* is associated with reports of both beneficial and pathogenic effects on human health (Ezeji et al., 2021). *Parabacteroides goldsteinii* ameliorates COPD. Lipopolysaccharides extracted from *Parabacteroides goldsteinii* have anti-inflammatory function and significantly improve COPD as an antagonist of the toll-like receptor 4 signaling pathway (Lai et al., 2022).

The microbiota may contribute to disease susceptibility *via* metabolite-mediated immunological progression (Segal et al., 2017). PICRUSt2 based on KEGG hierarchy (level 3) revealed that atopic wheezing-associated differences in predicted bacterial functions included cytoskeleton proteins, glutamatergic synapses, and porphyrin and chlorophyll metabolism pathways. This indicated that functional differences in respiratory microbiota existed between the Aw and nAw groups. It has been reported that the cytoskeleton plays a key regulatory role in maintaining the cell barrier. The increased permeability of epithelial and endothelial cells is mainly due to the reorganization of cytoskeletal junction complexes. Proinflammatory mediators, such as cytokines, can induce cytoskeleton rearrangement, leading to inflammation-dependent intestinal barrier function defects (López-Posadas et al., 2017). Secreted gastrointestinal microbial factors induce EMT-like properties in tumor cells, and thus promote disease progression by affecting cytoskeletal reorganization (Henstra et al., 2021). Lung bacteria contain greater amounts of cytoskeletal proteins and have greater function in energy production and conversion compared to intestinal bacteria (Liu et al., 2020a). Previous studies have revealed that fecal microbiota transplantation might be an efficient way to improve the physiology and behavior of chickens by increasing the functional capabilities of microbial communities in glutamatergic synapse-related pathways (Yan et al., 2021). Porphyrin and chlorophyll metabolism were significantly enriched in allergic rhinitis patients (Liu et al., 2020b), but were reduced in children with chronic pancreatitis when predictive metagenomic functional analysis was conducted (Wang et al., 2020). So far, there are only limited reports about this bacterial functional pathway in humans, and its role in wheezing and asthmatic children remains to be further elucidated.

This study has some limitations. The sample size was on the smaller side; therefore, a larger sample size is required to confirm the accuracy of our results. In addition, for ethical reasons, healthy infants could not be used as a control group, which may affect the validity of the results. The study mainly focused on bacterial abundance rather than viral and fungal studies. Viral respiratory infections are proposed to be the main cause of recurrent wheezing in infants (Parisi et al., 2022). Future studies utilizing systems biology approaches to assess bacterial, viral, and fungal microbiomes would help us better understand host-microbiota interactions and their significance in recurrent wheezing in infants. Finally, this was a cross-sectional observational study, and further longitudinal and unbiased studies of airway microbiota analysis will be useful for understanding the mechanisms underlying recurrent wheezing in infants.

## 5 Conclusion

In conclusion, the differential candidate biomarkers identified by microbiome analysis in our work may have reference value for the diagnosis of wheezing in infants with atopy. To confirm that, airway microbiome combined with metabolomics analysis should be further investigated in the future.

## Data availability statement

The data presented in the study are deposited in the Mendeley repository, accession link: dx.doi.org/10.17632/d4kb7zy533.1.

## Ethics statement

This study was approved by the Ethics Review Board of Chengdu Women’s and Children’s Central Hospital 2016 (22). Written informed consent was obtained from the guardians of all participants.

## Author contributions

WT and LZ came up with the original idea. WT, LZ, and TA performed the BAL collection and 16S rRNA gene sequencing. WT, WX, CX, and YF carried out the data analysis. WT wrote the first version of the manuscript. SC, ZC, JY, and YP made corresponding contributions to the paper. All listed authors made substantial, direct, and intellectual contributions to the work and approved its publication.
